# Personalized intraoperative arterial pressure management and mitochondrial oxygen tension in patients having major non-cardiac surgery: a pilot substudy of the IMPROVE trial

**DOI:** 10.1007/s10877-024-01260-0

**Published:** 2025-02-07

**Authors:** Moritz Flick, Christina Vokuhl, Alina Bergholz, Kristina Boutchkova, Julia Y. Nicklas, Bernd Saugel

**Affiliations:** 1 Department of Anesthesiology, Center of Anesthesiology and Intensive Care Medicine, University Medical Center Hamburg-Eppendorf, Martinistrasse 52, 20246 Hamburg, Germany; 2 Outcomes Research Consortium, Cleveland, OH USA

**Keywords:** Anesthesia, Cardiovascular dynamics, Hemodynamic monitoring, Individualized, Microcirculation, Tissue perfusion

## Abstract

The “Cellular Oxygen METabolism” (COMET) system (Photonics Healthcare, Utrecht, The Netherlands) non-invasively measures mitochondrial oxygen tension (mitoPO_2_) in the skin. The effects of general anesthesia and major non-cardiac surgery on mitoPO_2_ remain unknown. In this pre-planned pilot substudy of the “Intraoperative blood pressure Management based on the individual blood PRessure profile: impact on postOperatiVE organ function” (IMPROVE) trial, we measured mitoPO_2_ from induction of general anesthesia until the end of surgery in 19 major non-cardiac surgery patients (10 assigned to personalized and 9 to routine intraoperative arterial pressure management). In the overall cohort, the median (25th to 75th percentile) preoperative awake mitoPO_2_ was 63 (53 to 82) mmHg and mitoPO_2_ after induction of general anesthesia was 42 (35 to 59) mmHg. The intraoperative average mitoPO_2_ was 39 (30 to 50) mmHg. Thirteen patients (68%) had intraoperative mitoPO_2_ values below 20 mmHg and the median percentage of surgical time with mitoPO_2_ < 20 mmHg was 17 (0 to 31)%. MitoPO_2_ was weakly correlated with mean arterial pressure (repeated measures correlation (r_rm_(*n*); r_rm_(984) = 0.26, 95% confidence interval 0.20 to 0.32; P < 0.001), but not meaningfully with heart rate (r_rm_(984) = -0.05, 95% confidence interval -0.11 to 0.01; P = 0.117). There was no important difference in intraoperative average mitoPO_2_ between patients assigned to personalized or to routine intraoperative arterial pressure management (P = 0.653). MitoPO_2_ under general anesthesia was about a quarter lower than preoperative awake mitoPO_2_, substantially fluctuated during major non-cardiac surgery, and transiently decreased below 20 mmHg in about two-thirds of the patients. Personalized – compared to routine – intraoperative arterial pressure management did not increase intraoperative mitoPO_2_. Whether intraoperative decreases in mitoPO_2_ are clinically meaningful warrants further investigation.

**Supplementary Information:**

The online version contains supplementary material available at 10.1007/s10877-024-01260-0.

## Introduction

Intraoperative hemodynamic management aims at optimizing arterial pressure and blood flow to ensure adequate organ perfusion and tissue oxygenation [1]. Tissue oxygenation is determined by oxygen delivery and cellular oxygen uptake [2]. However, it is not yet possible to routinely monitor cellular oxygen uptake during surgery [3].

About 90% of cellular oxygen is shifted to mitochondria for cellular respiration [4]. The “Cellular Oxygen METabolism” (COMET) system (Photonics Healthcare, Utrecht, The Netherlands) non-invasively measures mitochondrial oxygen tension (mitoPO_2_) in the skin using the protoporphyrin IX-triplet state lifetime technique [5, 6]. In short, mitochondrial oxygen content correlates inversely with fluorescence of protoporphyrin IX [7], which can be enriched in the skin by topical application of 5-aminolevulinic acid [8]. The COMET system measures the fluorescence of protoporphyrin IX to calculate mitoPO_2_. Monitoring mitoPO_2_ could potentially help clinicians guide intraoperative hemodynamic management. However, it is unknown if mitoPO_2_ is impaired during non-cardiac surgery with general anesthesia and if it is affected by intraoperative hemodynamic management.

In this pre-planned pilot substudy of the “Intraoperative blood pressure Management based on the individual blood PRessure profile: impact on postOperatiVE organ function" (IMPROVE) trial [9], we thus aimed to investigate the effects of general anesthesia and major non-cardiac surgery on mitoPO_2_. We further aimed to investigate if personalized – compared to routine – intraoperative arterial pressure management increases intraoperative mitoPO_2_ in patients having major non-cardiac surgery.

## Materials and methods

### Study design

This pre-planned pilot substudy of the IMPROVE trial [9] was conducted between July 2018 and April 2019 in the Department of Anesthesiology, Center of Anesthesiology and Intensive Care Medicine, University Medical Center Hamburg-Eppendorf (Hamburg, Germany). The IMPROVE trial including this pilot substudy was approved by the ethics committee (Ethikkommission der Ärztekammer Hamburg, Hamburg, Germany) on December 20, 2016 (registration number PV5413). All patients provided written informed consent. The IMPROVE trial tested the primary hypothesis that personalized intraoperative arterial pressure management maintaining preoperative baseline mean arterial pressure (MAP) from automated 24-h arterial pressure monitoring reduces the incidence of neurocognitive disorders (composite of delayed neurocognitive recovery and delirium) between postoperative days 3 and 7 compared to routine intraoperative arterial pressure management in patients having elective major non-cardiac surgery.

### Patients

The IMPROVE trial included patients scheduled for elective major non-cardiac surgery with general anesthesia expected to last ≥ 90 min who were ≥ 50 years old and classified as American Society of Anesthesiologists physical status class II–IV. Patients who had a history of cerebrovascular events; had dementia; had kidney transplants; required dialysis; or were scheduled for emergency, cardiac, vascular, transplant, or neurosurgery were not included in the trial.

### Basic anesthetic management

Epidural catheters – if clinically indicated – were inserted before induction of general anesthesia. General anesthesia was induced with sufentanil, propofol, and rocuronium or cisatracurium boluses according to routine care. After endotracheal intubation, the patients’ lungs were mechanically ventilated with a target end-tidal carbon dioxide of 35–40 mmHg. To maintain general anesthesia, patients received continuous remifentanil infusion or repeated boluses of sufentanil with inhaled sevoflurane or continuous propofol infusion. During surgery, arterial pressure was measured using a radial artery catheter.

### Protocol

In the IMPROVE trial, patients were randomized in a 1:1 ratio to personalized or to routine intraoperative arterial pressure management. All patients had preoperative automated 24-h arterial pressure monitoring. Preoperative baseline MAP was individually defined as the mean of the mean daytime MAP and mean nighttime MAP measurements. In patients assigned to personalized intraoperative arterial pressure management, clinicians were asked to maintain intraoperative MAP above the preoperative baseline MAP. Specifically, clinicians were asked to maintain MAP within a range between the preoperative baseline MAP + 10 mmHg (with a minimum MAP target range of 65–75 mmHg and a maximum MAP target range of 100–110 mmHg). In patients assigned to routine intraoperative arterial pressure management, clinicians managed arterial pressure per institutional routine which generally is to maintain intraoperative MAP above 65 mmHg [10, 11]. Norepinephrine was used as the primary vasopressor per institutional routine. For the analysis, MAP values were recorded every five minutes during the induction of general anesthesia and surgery.

### Measurement of mitoPO_2_

MitoPO_2_ was measured using the COMET system. On the evening before surgery, a 4 cm^2^ patch containing 8 mg 5-aminolevulinic acid (Alacare; Photonamic, Pinneberg, Germany) was applied to the clavipectoral triangle or above the sternum and protected from light with an additional patch. On the following day, before induction of general anesthesia, the patches were removed, the skin was cleaned, and the sensor of the COMET system was attached on the prepared skin area and shielded from surrounding light. We started measuring mitoPO_2_ with the COMET system before induction of general anesthesia and continued the measurements until the end of surgery. The COMET system provides updated mitoPO_2_ values every minute and additionally records the sensor temperature on the skin. We removed artefactual measurements before the analysis.

### Endpoints

We calculated 5-min averages of mitoPO_2_ before induction of general anesthesia (preoperative awake mitoPO_2_), after induction of general anesthesia, at the beginning of surgery, and at the end of surgery. We further calculated average mitoPO_2_ during induction of general anesthesia and average mitoPO_2_ during surgery. Further, we report the lowest mitoPO_2_ and the lowest average mitoPO_2_ for 5 continuous minutes, during induction of general anesthesia and during surgery as well as the percentage of surgical time with mitoPO_2_ < 50 mmHg, < 40 mmHg, < 30 mmHg, and < 20 mmHg. All endpoints were calculated for the overall cohort and separately for patients assigned to personalized or to routine intraoperative arterial pressure management. Finally, we analyzed the correlation between mitoPO_2_ and MAP as well as the correlation between mitoPO_2_ and heart rate. On an exploratory basis, we compared mitoPO_2_ values between patients assigned to personalized and to routine intraoperative arterial pressure management.

### Statistical analysis

Baseline, and clinical characteristics are reported separately for patients assigned to personalized and to routine intraoperative arterial pressure management. Categorical data are presented as absolute number (percentage), and continuous data are presented as median (25th to 75th percentile). We compared measurements between time points using two-tailed Wilcoxon signed-rank tests and measurements between patients assigned to personalized and to routine intraoperative arterial pressure management using two-tailed Mann–Whitney U-tests. The correlation between mitoPO_2_ and MAP as well as the correlation between mitoPO_2_ and heart rate were assessed using repeated measures correlation (r_rm_(*n*)) with 95%-confidence intervals (95% CI) [12, 13].

P-values are presented as descriptive summary measures, are not adjusted for multiple testing, and should not be interpreted as results of confirmatory hypothesis testing. Data were analyzed using GraphPad Prism V 10.1.1 (GraphPad Software, Boston, USA).

In the absence of previous studies investigating mitoPO_2_ in patients having non-cardiac surgery, no formal sample size calculation was performed for this pilot substudy. The observed mean difference in mitoPO_2_ before and after induction of general anesthesia of 20 mmHg (standard deviation 19 mmHg) in 19 patients corresponds to an effect size of −1.05 and results in a power (1-β error probability) of 98.8% with an α error probability of 0.05. The power calculation was performed using G*Power 3.1.9.7 (Kiel University, Germany) [14]. We included IMPROVE patients depending on the availability of study personnel and the COMET system.

## Results

**Fig. 1:**
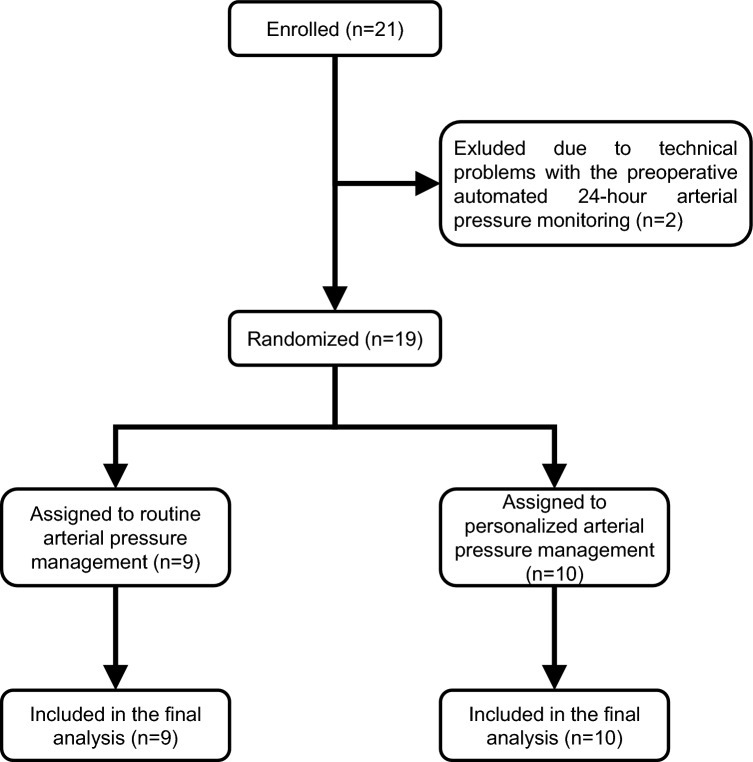
Flow chart illustrating enrolment, randomization, and reasons for exclusion

We included 21 patients in this pilot substudy, but excluded two who were excluded from the IMPROVE trial before randomization because of technical problems with the automated 24-h arterial pressure monitoring (Fig. 1; Table 1).

**Table 1:** Patient characteristics

	Overall (n = 19)	Routine intraoperative arterial pressure management (n = 9)	Personalized intraoperative arterial pressure management (n = 10)
Age, years	64 (61 to 71)	64 (60 to 72)	65 (61 to 70)
Female, n	9 (47%)	5 (56%)	4 (40%)
Height, cm	172 (164 to 176)	168 (165 to 172)	174 (166 to 182)
Weight, kg	77 (69 to 93)	77 (70 to 90)	77 (65 to 93)
Body mass index, kg/m^2^	25.7 (22.9 to 30.4)	29.3 (24.0 to 31.0)	24.2 (22.3 to 26.2)
American Society of Anesthesiologists physical status, II/III/IV	7/11/1	3/6/0	4/5/1
Arterial hypertension, n	13 (68%)	6 (67%)	7 (70%)
Chronic obstructive pulmonary disease, n	1 (5%)	1 (11%)	0 (0%)
Coronary artery disease, n	3 (16%)	2 (22%)	1 (10%)
Diabetes mellitus, n	2 (11%)	1 (11%)	1 (10%)
Type of surgery, n			
*General*	8 (42%)	4 (44%)	4 (40%)
*Urology*	6 (32%)	2 (22%)	4 (40%)
*Gynecology*	3 (16%)	2 (22%)	1 (10%)
*Trauma/Orthopedic*	2 (11%)	1 (11%)	1 (10%)
Epidural catheter, n	14 (74%)	6 (67%)	8 (80%)

Data are presented as median (25th to 75th percentile) or absolute frequency (percentage) as appropriate

**Fig. 2:**
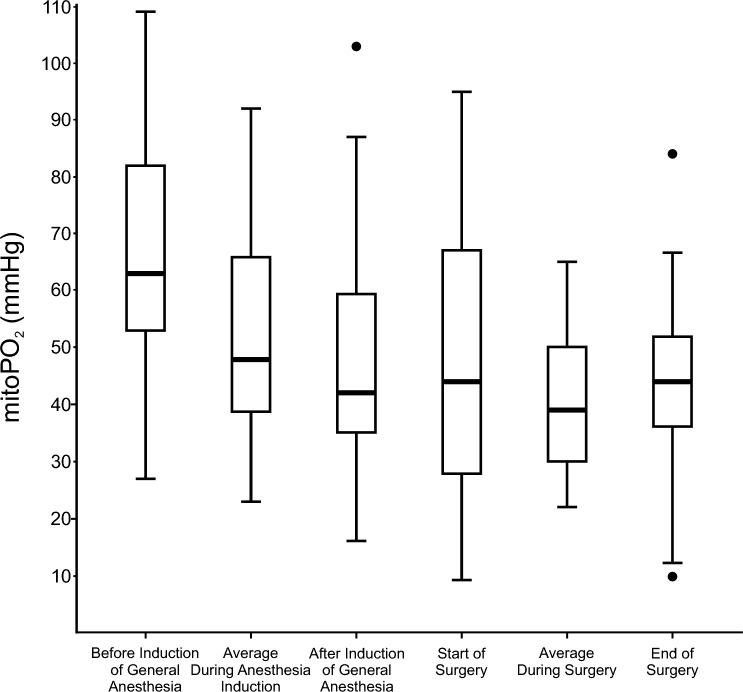
Box plots showing mitochondrial oxygen tension (mitoPO_2_). The central box represents the values from the 25th to 75th percentile and its middle line represents the median. The vertical line extends from the minimum to the maximum value, excluding outliers. Outliers are defined as values that are smaller than the lower quartile minus 1.5 times or larger than the upper quartile plus 1.5 times the interquartile range

In the overall cohort, the median (25th to 75th percentile) preoperative awake mitoPO_2_ was 63 (53 to 82) mmHg before induction of general anesthesia (Fig. 2, Supplementary Table 1). After induction of general anesthesia, the median mitoPO_2_ was 42 (35 to 59) mmHg. The mitoPO_2_ after induction of general anesthesia was lower than the preoperative awake mitoPO_2_ in all but two patients (median difference −22 (−32 to −5) mmHg or −23 (−45 to −10) %; P < 0.001). During induction of general anesthesia, the median average mitoPO_2_ was 48 (38 to 66) mmHg, the median lowest mitoPO_2_ was 31 (17 to 46) mmHg, and the median lowest average mitoPO_2_ for 5 continuous minutes was 37 (22 to 50) mmHg.

During surgery, the median average mitoPO_2_ was 39 (30 to 50) mmHg (Fig. 2; Table 2). The median mitoPO_2_ levels at the beginning of surgery (44 (28 to 64) mmHg) were similar to those at the end of surgery (44 (36 to 52) mmHg). However, in 13 patients (68%), the difference between mitoPO_2_ values at the beginning and the end of surgery exceeded ± 10 mmHg – increasing in 7 patients (37%) and decreasing in 6 patients (32%). During surgery, the median lowest mitoPO_2_ was 5 (4 to 7) mmHg and the median lowest average mitoPO_2_ for 5 continuous minutes was 9 (5 to 23) mmHg. In only two patients (11%) all intraoperative mitoPO_2_ was always higher than 50 mmHg. Thirteen patients (68%) had intraoperative mitoPO_2_ values below 20 mmHg. The median percentage of surgical time with mitoPO_2_ < 20 mmHg was 17 (0 to 31) %.

**Table 2:** Intraoperative mitoPO_2_

	Overall (n = 19)	Routine intraoperative arterial pressure management (n = 9)	Personalized intraoperative arterial pressure management (n = 10)	*P-value*
Average intraoperative mitoPO_2_, mmHg	39 (30 to 50)	46 (30 to 51)	38 (31 to 44)	0.653
Lowest intraoperative mitoPO_2_, mmHg	7 (5 to 20)	14 (6 to 22)	5 (4 to 7)	0.072
Lowest intraoperative 5-min average mitoPO_2_, mmHg	9 (7 to 26)	17 (8 to 24)	9 (5 to 23)	0.390
Percentage of surgical time with mitoPO_2_ < 50 mmHg, %	81 (48 to 90)	85 (52 to 93)	78 (52 to 87)	0.772
Percentage of surgical time with mitoPO_2_ < 40 mmHg, %	43 (21 to 73)	35 (20 to 73)	46 (29 to 73)	0.810
Percentage of surgical time with mitoPO_2_ < 30 mmHg, %	28 (7 to 55)	23 (7 to 45)	35 (10 to 56)	0.596
Percentage of surgical time with mitoPO_2_ < 20 mmHg, %	17 (0 to 31)	4 (0 to 31)	17 (4 to 25)	0.711

Data are presented as median (25th to 75th percentile). P-values reflect the difference between patients assigned to routine and personalized intraoperative arterial pressure management. *mitoPO*_*2*_* – mitochondrial oxygen tension*

**Fig. 3:**
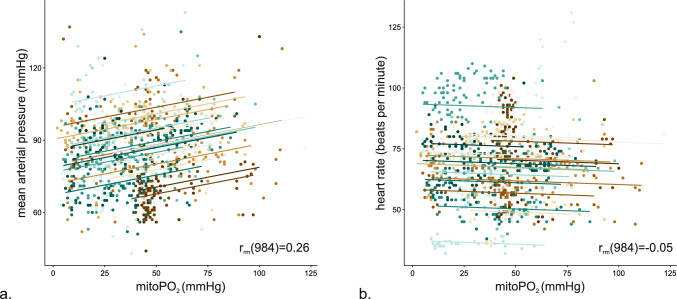
Repeated measures correlation between mitochondrial oxygen tension (mitoPO_2_) and mean arterial pressure and between mitoPO_2_ and heart rate. Colored dots reflect measurement pairs from individual patients. Colored lines indicate the overall regression line with data points from individual patients

The correlation between mitoPO_2_ and MAP was weak (r_rm_(984) = 0.26, 95% CI 0.20 to 0.32; P < 0.001). There was no meaningful correlation between mitoPO_2_ and heart rate (r_rm_(984) = −0.05, 95% CI −0.11 to 0.01; P = 0.117) (Fig. 3).

In patients assigned to personalized intraoperative arterial pressure management, the median intraoperative average MAP (92 (88 to 102) mmHg vs. 81 (71 to 90) mmHg; P = 0.008) and the median average norepinephrine infusion rate (0.22 (0.13 to 0.33) µg/kg/min vs. 0.06 (0.05 to 0.19) µg/kg/min; P = 0.015) were higher than in patients assigned to routine intraoperative arterial pressure management (Table 3). The cumulative amount of crystalloids and colloids patients were given was similar between the two groups (Table 3).

**Table 3:** Intraoperative data

	Overall (n = 19)	Routine intraoperative arterial pressure management (n = 9)	Personalized intraoperative arterial pressure management (n = 10)	*P-value*
Duration of surgery, min	230 (130 to 330)	170 (125 to 300)	248 (181 to 348)	0.289
Crystalloids, ml	2500 (2000 to 4500)	2500 (1500 to 4000)	3500 (2125 to 4500)	0.389
Colloids, ml	50 (0 to 1500)	0 (0 to 1500)	500 (0 to 1375)	0.653
Norepinephrine infusion rate, µg/kg/min	0.15 (0.07 to 0.24)	0.06 (0.05 to 0.19)	0.22 (0.13 to 0.33)	0.014
Blood loss, ml	500 (315 to 1100)	315 (175 to 725)	800 (600 to 1100)	0.749
Preoperative baseline MAP, mmHg	96 (91 to 99)	96 (86 to 98)	97 (93 to 103)	0.390
Intraoperative MAP, mmHg	89 (81 to 94)	81 (73 to 89)	92 (89 to 101)	0.178
Intraoperative heart rate, beats/minute	70 (63 to 80)	77 (63 to 81)	69 (64 to 74)	0.490

Data are presented as median (25th to 75th percentile). P-values reflect the difference between patients assigned to routine and personalized intraoperative arterial pressure management. *MAP – mean arterial pressure*

During surgery, the median average mitoPO_2_ was 38 (31 to 44) mmHg in patients assigned to personalized and 46 (30 to 51) mmHg in patients assigned to routine intraoperative arterial pressure management (P = 0.653). There was also no clinically meaningful difference in the lowest intraoperative mitoPO_2_, the lowest average mitoPO_2_ for 5 continuous minutes, and the percentage of surgical time with mitoPO_2_ < 50 mmHg, < 40 mmHg, < 30 mmHg, and < 20 mmHg between patients assigned to personalized or to routine arterial pressure management (Table 2).

## Discussion

In this pilot substudy of the IMPROVE trial, mitoPO_2_ under general anesthesia was about a quarter lower than preoperative awake mitoPO_2_ in patients having major non-cardiac surgery. During surgery, mitoPO_2_ substantially fluctuated and transiently decreased below 20 mmHg in about two-thirds of the patients. Personalized – compared to routine – intraoperative arterial pressure management did not increase intraoperative mitoPO_2_.

In our study, the mitoPO_2_ values before induction of general anesthesia were similar to those found in previous studies in young healthy volunteers [15, 16]. In line with these previous studies, we also found that mitoPO_2_ substantially varies among individuals. MitoPO_2_ under general anesthesia was about a quarter lower than preoperative awake mitoPO_2_ – whether this is the result of reduced oxygen delivery or altered oxygen utilization remains unknown. However, previous observational studies have shown that energy expenditure under general anesthesia is about one-quarter lower than preoperative awake resting energy expenditure in patients having non-cardiac surgery [17, 18].

The intraoperative average mitoPO_2_ values in our major abdominal surgery patients were similar to those reported in a study of 20 patients having neurosurgery [19]. However, in these neurosurgical patients, mitoPO_2_ decreased from around 60 mmHg to around 40 mmHg over the course of surgery. In our study, individual mitoPO_2_ substantially fluctuated and transiently decreased below 20 mmHg in about two-thirds of the patients. These mitoPO_2_ fluctuations occurred mostly independent of macrohemodynamic alterations and the overall correlation between mitoPO_2_ and MAP was weak. It will require investigation in larger cohorts, whether or not transient – short or prolonged – decreases in mitoPO_2_ are clinically important.

We also investigated the effect of two different intraoperative arterial pressure management strategies on mitoPO_2_. Personalized – compared to routine – intraoperative arterial pressure management resulted in higher MAPs, but there was no clinically important difference in intraoperative mitoPO_2_ between the two groups. Profound hypotension can lead to tissue hypoperfusion – and low mitoPO_2_ values. However, profound hypotension (with MAP values substantially below 65 mmHg) was rare in both groups. The few episodes of intraoperative hypotension we observed were short and not associated with low mitoPO_2_ values. Patients assigned to personalized intraoperative arterial pressure management were given more norepinephrine. Interestingly, these higher norepinephrine doses, that were required to maintain MAP in patients assigned to personalized intraoperative arterial pressure management also did not decrease mitoPO_2_.

MitoPO_2_ measurements in our study were feasible and safe. We did not observe any adverse events associated with mitoPO_2_ measurements. Monitoring mitoPO_2_ with the protoporphyrin IX triplet state lifetime method is an innovative and intriguing concept as it provides insight into the final pathway of oxygen delivery to the cells. Assuming that elective non-cardiac surgery patients do not have mitochondrial dysfunction or severe impairment of oxygen utilization, mitoPO_2_ could be a better surrogate for oxygen delivery and cellular oxygen uptake than estimation from systemic oxygen delivery or monitoring regional oxygen saturation with near-infrared spectroscopy – which measure oxygen content in the blood rather than in the tissue [20]. However, until now, monitoring mitoPO_2_ is only possible on the skin – which may not necessarily reflect mitoPO_2_ of vital organs. Changes in cutaneous mitoPO_2_ may thus not always mirror changes in visceral mitoPO_2_. Even though we carefully prepared the skin and carefully performed mitoPO_2_ measurements, it cannot be ruled out that there were constraining factors affecting the mitoPO_2_ measurements during surgery. Whether or not monitoring mitoPO_2_ in the skin provides clinically meaningful information and can help guide perioperative hemodynamic management in high-risk patients requires further investigation.

This was a pilot substudy of the monocentric IMPROVE trial. Due to the complexity of the mitoPO_2_ measurements, we only included a few patients. Our results thus need to be confirmed in larger studies. Naturally, the results are not generalizable to other settings, e.g., patients in the emergency room or the intensive care unit. We did not record the fraction of inspired oxygen, which may have an important effect on mitoPO_2_. Further, we only measured heart rate and arterial pressure as macrocirculatory variables and thus were not able to analyze the relationship between blood flow and mitoPO_2_.

MitoPO_2_ under general anesthesia was about a quarter lower than preoperative awake mitoPO_2_, substantially fluctuated during major non-cardiac surgery, and transiently decreased below 20 mmHg in about two-thirds of the patients. Personalized – compared to routine – intraoperative arterial pressure management did not increase intraoperative mitoPO_2_. Whether intraoperative decreases in mitoPO_2_ are clinically meaningful warrants further investigation.

## Supplementary Information

Below is the link to the electronic supplementary material.

Supplementary file1 (PDF 249 KB)

## Data Availability

Data are available upon reasonable request.
