# Supplementary material for: Personalized intraoperative arterial pressure management and mitochondrial oxygen tension in patients having major non-cardiac surgery: a pilot substudy of the IMPROVE trial
Source: J Clin Monit Comput. 2025 Feb 7;39(5):813–20. doi: 10.1007/s10877-024-01260-0 (PMC12474711; doi:10.1007/s10877-024-01260-0)
Supplement: Supplementary file 1 — Supplementary file1 (PDF 249 KB) [file 10877_2024_1260_MOESM1_ESM.pdf]

Supplementary Table 1 mitoPO_2 during induction of anesthesia

	**Overall (n=19)**	**Routine blood pressure management (n=9)**	**Personalized blood pressure management (n=10)**	***P-value***
Preoperative awake mitoPO2, mmHg	63 (53 to 82)	61 (44 to 69)	77 (58 to 96)	0.205
Average intraoperative mitoPO_2, mmHg	48 (38 to 66)	46 (35 to 59)	57 (52 to 71)	0.327
mitoPO_2 after anesthesia induction, mmHg	42 (35 to 59)	37 (34 to 58)	47 (37 to 67)	0.368
Lowest mitoPO_2 during anesthesia induction, mmHg	31 (17 to 46)	29 (18 to 49)	39 (16 to 45)	0.936
Lowest mitoPO_2 for 5 continuous minutes during anesthesia induction, mmHg	37 (22 to 50)	34 (27 to 54)	43 (21 to 46)	0.741

Data are presented as median (25th to 75th percentile). P-values reflect the difference between patients assigned to routine blood pressure management and personalized blood pressure management. *mitoPO_2 – mitochondrial oxygen tension*
